# Is There a Role for Combined EMG-fMRI in Exploring the Pathophysiology of Essential Tremor and Improving Functional Neurosurgery?

**DOI:** 10.1371/journal.pone.0046234

**Published:** 2012-10-01

**Authors:** Maria Fiorella Contarino, Paul F. C. Groot, Johan N. van der Meer, Lo J. Bour, Johannes D. Speelman, Aart J. Nederveen, Pepijn van den Munckhof, Marina A. J. Tijssen, Peter Rick Schuurman, Anne-Fleur van Rootselaar

**Affiliations:** 1 Department of Neurology/Clinical Neurophysiology, Academic Medical Center, Amsterdam, The Netherlands; 2 Department of Radiology, Academic Medical Center, Amsterdam, The Netherlands; 3 Department of Neurosurgery Academic Medical Center, Amsterdam, The Netherlands; Centre Hospitalier Universitaire Vaudois Lausanne - CHUV, UNIL, Switzerland

## Abstract

**Background:**

Functional MRI combined with electromyography (EMG-fMRI) is a new technique to investigate the functional association of movement to brain activations. Thalamic stereotactic surgery is effective in reducing tremor. However, while some patients have satisfying benefit, others have only partial or temporary relief. This could be due to suboptimal targeting in some cases. By identifying tremor-related areas, EMG-fMRI could provide more insight into the pathophysiology of tremor and be potentially useful in refining surgical targeting.

**Objective:**

Aim of the study was to evaluate whether EMG-fMRI could detect blood oxygen level dependent brain activations associated with tremor in patients with Essential Tremor. Second, we explored whether EMG-fMRI could improve the delineation of targets for stereotactic surgery.

**Methods:**

Simultaneous EMG-fMRI was performed in six Essential Tremor patients with unilateral thalamotomy. EMG was recorded from the trembling arm (non-operated side) and from the contralateral arm (operated side). Protocols were designed to study brain activations related to voluntary muscle contractions and postural tremor.

**Results:**

Analysis with the EMG regressor was able to show the association of voluntary movements with activity in the contralateral motor cortex and supplementary motor area, and ipsilateral cerebellum. The EMG tremor frequency regressor showed an association between tremor and activity in the ipsilateral cerebellum and contralateral thalamus. The activation spot in the thalamus varied across patients and did not correspond to the thalamic nucleus ventralis intermedius.

**Conclusion:**

EMG-fMRI is potentially useful in detecting brain activations associated with tremor in patients with Essential Tremor. The technique must be further developed before being useful in supporting targeting for stereotactic surgery.

## Introduction

### Essential Tremor (ET) and Stereotactic Surgery

Available studies on the pathophysiology of tremor in ET show that the cerebellum and the cerebellothalamocortical pathway are involved in tremor generation [1]–[5].

Stereotactic ablation and stimulation of the nucleus ventralis intermedius of the thalamus (Vim) effectively suppress tremor, suggesting a crucial role of this nucleus in the pathophysiology of tremor. However, outcome of surgery for ET is variable: while some patients have satisfying and long-lasting benefit, others have only partial or temporary benefit [6]–[9]. This variability in clinical outcome might be due to differences in the position of the lesion (or electrode) or to inter-individual differences in the location of the tremor-related areas. Alternative targets have been also used for the treatment of tremor, with good results. These include brain nuclei either within the thalamus (ventro-oralis anterior (Voa), ventro-oralis posterior (Vop)) or outside it (*zona incerta*, Forel fields, posterior subthalamic area, subthalamic nucleus [10]–[16]). Pre-operative non-invasive identification of tremor-related areas for each individual patient might help surgical teams refining their stereotactic target.

### EMG-fMRI

Functional Magnetic Resonance Imaging (fMRI) is used to study brain activations in relation to a task, based on blood oxygen level-dependent (BOLD) contrasts. During a typical block design, tasks are performed in an on/off fashion (boxcar type), which allows comparing brain activity during a task with rest, or different conditions. This kind of design gives no control on possible mistakes in task performance, like delays in starting/ending the tasks or inconstant quality of the performance.

Simultaneous recording of surface electromyography (EMG) and fMRI allows monitoring of motor task execution, but also accurate time-locking of the presence and intensity of tremor to the functional imaging. Since movement disorders often occur simultaneously to voluntary movements, it is almost impossible to distinguish brain activity related to the voluntary task (e.g. maintaining posture) from activity related to the involuntary movement (e.g. postural tremor). However, amplitude variations of the EMG, after Gram-Schmidt orthogonalization with respect to the motor task, are supposed to reflect the extra EMG activity induced by tremor. [17], [18] As opposed to conventional fMRI analysis based on block design, by using these amplitude variations as regressors in the EMG-fMRI analysis, one could more reliably investigate the relation between tremor-related EMG information and BOLD activity in the brain. This approach has been successfully used to study different kinds of tremor, like cortical tremor [17], [19] and parkinsonian rest tremor [18], but it has never been used to study ET tremor. Other studies have investigated the pathophysiology of ET or the changes in brain activity associated with DBS of the thalamus, but with other imaging modalities such as positron emission tomography (PET) [20], Magnetoencephalography (MEG) [21], or EMG-EEG coherence [22].

The aim of the present study was twofold. First, we wanted to provide a proof of principle of the ability of combined EMG-fMRI to detect BOLD activations in brain areas associated with tremor in ET patients, by using EMG parameters as regressors in the analysis. We hypothesized that EMG-fMRI would show areas known to be involved with the pathogenesis of tremor (basal ganglia, cerebellum and thalamus).

Secondly, we wanted to test whether EMG-fMRI could predict the best thalamic spot to be targeted for stereotactic surgery. For this purpose, we selected a group of ET patients with prior successful unilateral Vim thalamotomy, as defined by a persistent reduction of at least 2 points on the tremor score of the right arm. This group of patients presents unique characteristics: it is a very homogeneous group of patients with similar clinical features of advanced, medication-resistant disease. In addition, by comparing the operated side with the non-operated side, each patient can serve as his/her own control. We hypothesized that EMG-fMRI would detect an active area in the non-operated thalamic region associated to tremor in the contralateral arm. In case of successful thalamotomy, this tremor-related area would correspond to the target area lesioned in the operated thalamus. Ultimately, EMG-fMRI could be used to predict the best surgical target for each individual patient and improve the benefit of stereotactic neurosurgery for tremor.

## Methods

### Ethics Statement

All procedures were carried out with the adequate understanding and written informed consent of the subjects. Reduced capacity/ability to consent, as assessed by a neurologist involved in the study, was considered as exclusion criterion from the study. The Medical Ethical Committee of the Academic Medical Center of Amsterdam gave approval to the study.

### Patients

From 1990 to 2008, 25 ET patients underwent unilateral thalamotomy at our center. Four patients died, nine had contralateral electrodes for thalamic stimulation, six declined participation due to poor general conditions and/or inability to reach the center. Six patients were included in the study. Demographical and clinical characteristics were collected from clinical files and patient interviews (Table 1). All the patients fulfilled the consensus criteria for definite classic ET [23], had disease duration longer than 5 years, had symptoms onset before the age of 65, and had at least one affected first-degree relative (“Hereditary ET”) [24]. In all patients symptoms were medication-resistant and severe enough to require surgery (ETRS item 5 score for the right arm was at least 6).

**Table 1:** Clinical and demographical characteristics of the patients included in the study, at the time of scanning.

Patient	1	2		3		4	5	6	Average±SD	
**Gender**	M	M		M		F	M	M		
**Age**	79	77		75		48	83	50	69±15	
**Age at onset**	64	51		16		6	14	43	32±23	
**Disease duration**	15	26		58		43	68	8	36±24	
**Thalamotomy side**	L	L		L		L	L	L		
**Dominant hand**	R	R		R		R	R	R		
**Time from thalamotomy (years)**	1	9		8		10	9	4 months	6±4	
**Total ETRS score at time of scanning**	19	64		67		23	37	20	38.3±22	
**Change in right arm tremor** **score** a	−5	na		−4		−7	−6	−3	−5	
**Right postural tremor score** b	0 P0 D	0 P0 D		0 P1 D		0 P1 D	1 P0 D	0 P0 D	0.2±0.40.3±0.5	
**Left postural Tremor score** b	0 P2 D	1 P2 D		1 P3 D		0 P2 D	1 P3 D	0 P1 D	0.5±0.52.2±0.8	
**Surgery side effects**	none	none		none		Transient dysarthria	Dysarthria, Babinski R, gait ataxia	none		
**Muscles used for EMG analysis**	EFL-EFR	FDIL-FDI R	EFL-BICEPS R		FDIL-ECR R		FDIL-FCRR	ECRL-FDI R		
**Tremor frequency**	4–7 Hz	3–6 Hz		2–6 Hz		4–8 Hz	3–7 Hz	3–6 Hz		

M, male; F, female; SD, standard deviation; ETRS, essential tremor rating scale;

a Change in total score of item 5 of the ETRS with respect to preoperative score (negative indicates improvement);

b Items 5 and 6 of the ETRS scored only with arms stretched, similarly to the scanning task; P, proximal; D, distal; EMG, electromyography, L, Left; R, right; EF, extensor of the finger; FDI, first dorsal interosseus; ECR, extensor carpi radialis; FCR, flexor carpi radialis; na, not available.

Patients discontinued anti-tremor medication the night before scanning. On the scanning day, the Essential Tremor Rating Scale (ETRS) was performed by an experienced movement disorders neurologist. Final Vim coordinates for each patient, based on preoperative targeting and adjusted after intraoperative test-stimulation, are given in Table S1. Further details on the methods are provided in Text S1.

### fMRI and EMG Acquisition

AT2*-weighted echo planar imaging (EPI) sequence and an isotropic T1-weighted 3D anatomical image were acquired on a 3-Tesla MRI scanner (Text S1). Two independent protocols were run. In total 160 EPI volumes for the “Movement” protocol and 240 EPI volumes for the “Tremor” protocol were acquired.

EMG data were recorded with an MRI-compatible EEG amplifier and EMG cable unit providing 16 Ag/AgCl surface electrodes configured for eight bipolar EMG derivations (Text S1). EMG was recorded at both arms from the extensor carpi radialis (ECR) and flexor carpi radialis (FCR) in all patients. Since the muscles in which tremor prevails vary among different patients and do not necessarily involve ECR and FCR, EMG data were recorded also from the two other arm muscles with strongest tremor in each patient. The choice of muscles was based on tremor intensity as revealed by clinical evaluation and inspection of EMG traces.

### Study Protocol

Two protocols were used, configured as a block design. The first (“Movement” protocol) was designed to detect the association of voluntary muscle contractions with brain areas known to be involved in voluntary movement. The active condition consisted of self-paced flexion-extension wrist movements performed while holding the arm stretched (left movement, LM; right movement, RM).

The second protocol (“Tremor” protocol) was designed to evaluate postural tremor. The active condition (maintained stretching of the arm with hand in pronation and extended wrist and fingers) was alternately performed with each arm (left stretching, LS; right stretching, RS) and with both arms together (both stretching, BS).

The active conditions were alternated with rest condition. Task and rest blocks lasted 29 s (10 EPI scans) in each protocol; each active condition was performed four times. The duration of the “Tremor” protocol was of about 11.5 minutes, and the duration of the “Movement” protocol was of about 8 minutes (total duration of the experimental protocol about 19.5 min). Patients could see instructions projected on a screen by an experimental control software (Presentation®, Neurobehavioral Systems Inc., www.neurobs.com), synchronized with the scanner. Patients practiced both protocols just before scanning and were instructed to hold arms and hands stretched such as to evoke tremor for the whole duration of the active conditions in the “Tremor” protocol. During execution of the task, patients were continuously monitored by an examiner to assure that performance was appropriate. Moreover, data on motor performance were continuously collected for all patients by means of EMG recordings (Figure S1).

### EMG Processing

Artifacts due to interaction with the magnetic field, and irregular volume- and slice-artifacts induced by limb motion, were removed with an fMRI artifact reduction algorithm [25]. The quality of the EMG signal after artifact correction was visually inspected. The signal was Hilbert-transformed, filtered, rectified [26], and transformed into frequency domain (Text S1). For the “Movement” protocol, we extracted frequencies from 1 to 250 Hz (Figure S2A.). The upper boundary of 250 Hz was chosen because there is generally no significant EMG power at higher frequencies [27]. Vectors were created from the absolute EMG power of the whole recordings of each muscle of the left and right arm.

**Figure 1:**
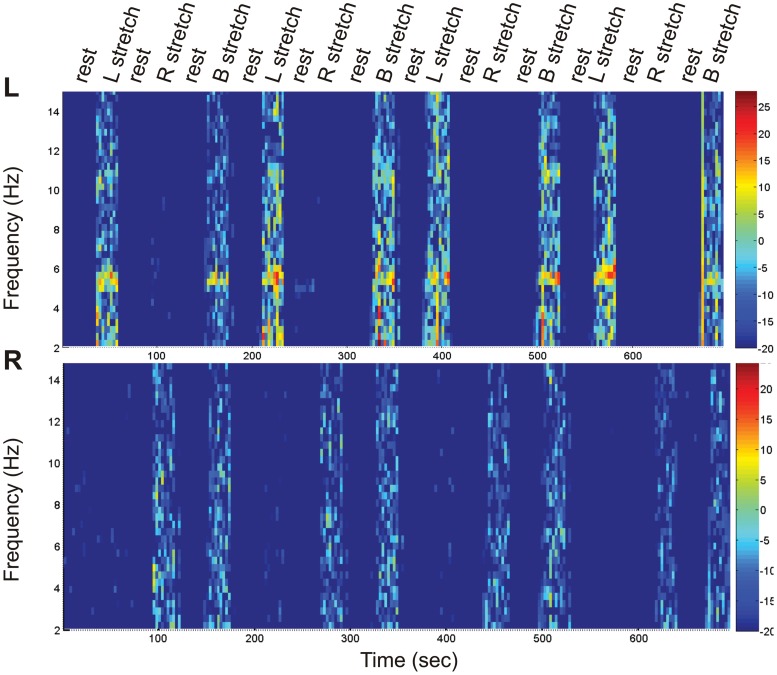
Spectrograms of EMG recorded during “Tremor” protocol. Spectrograms of the continuous simultaneous EMG recording from the Extensor of the fingers (EF) of the left (top panel) and right arm (bottom panel), during the conditions rest, left arm stretching (L stretch), right arm stretching (R stretch), and both arms stretching (B stretch) in patient No.1. The color bar on the right of the figure indicates power intensity going from low (deep blue) to high (red). A clear increase in the tremor frequency range is visible as an orange band between 4 and 6 Hz on the left EF spectrogram, during extension of the arms and fingers inducing postural tremor (conditions L stretch and B stretch). No increase in power in the tremor frequency is visible on the right EF spectrogram.

For the “Tremor” protocol, the tremor frequency was identified and extracted, based on individual frequency power spectra (Table 1, Figure 1). Three vectors were created from the EMG of each muscle: “EMG-LS”, consisting of the absolute EMG power during stretching of the left arm and zeros elsewhere, “EMG-RS” (stretching right arm), and “EMG-BS” (stretching of both arms). In order to make the EMG-derived vectors and the block-derived vectors independent, Gram-Schmidt orthogonalization was applied [28].

### fMRI Analysis

fMRI data were analyzed with the Statistical Parametric Mapping software (SPM8, Wellcome Department of Cognitive Neurology, London, UK; www.fil.ion.ucl.ac.uk/spm). The EMG was used to correct the block design with real onset/offset information (EMG-corrected block design). To correct for head movement, we excluded the functional scans that corresponded to acceleration greater than 0.4 mm/scan, by means of “scan-nulling” regressors [29]. EPI scans corresponding to residual MR artifacts on the EMG signal, especially at the beginning and end of active conditions, were also excluded by scan-nulling. Group analysis was performed on normalized images with fixed-effects approach (threshold of p<.001) [30].

#### “Movement” protocol

The whole vectors deriving from the HRF-convolved, non-orthogonalized, absolute power in the 1–250 Hz in the ECR muscle (EMG-Left and EMG-Right) were used as column vectors in the design, together with the scan-nulling regressors. The number of scan-nulled scans due to artifacts was on average 71 for the “movement” protocol.

For comparison, a second design was created with only the scan-nulling regressors and the block design, with three vectors: left arm movement (LM), right arm movement (RM), and rest, with onsets/offsets corrected based on the EMG.

For group analysis, brain activations were assessed using t-contrasts separately for EMG-Left and EMG-Right in the EMG design and for LM and RM in the Block design.

#### “Tremor” protocol

After inspection of the EMG, the muscle presenting most clear tremor activity was selected. When no tremor activity was present (as in some sides contralateral to thalamotomy) EMG with optimal artifact removal was selected. The condition BS, not useful for the aim of the present study, was excluded from the analysis by scan-nulling. Thus, the GLM design consisted of: a) The vectors deriving from the HRF-convolved and orthogonalized EMG power in the extracted tremor frequency range during LS for the selected muscle of the left arm (EMG-LS-Left) and during RS for right arm (EMG-RS-Right); b) the HRF-convolved, EMG-corrected block regressors encoding for the active conditions (LS and RS) and rest; c) regressors encoding scan-nulling, as covariates of no interest. The number of scan-nulled scans due to artifacts was on average 111 for the “tremor protocol”.

Brain activations were assessed using t-contrasts separately for EMG-LS-Left and EMG-RS-Right on a single-subject basis using a threshold of p<.001, to generate the first-level statistical activation maps (t-map). Functional images were smoothed using a 6×6×6 mm Gaussian kernel.

In consideration of the potential inter-subject anatomical variability, of the considerable degree of brain atrophy in some patients, and of the presence of the thalamotomy, and in order to enhance spatial accuracy, we chose not to normalize individual scans for the single-subject analysis. The functional t-maps were overlaid on each patient’s own T1 anatomical scan. As additional explorative analysis, group analysis was performed.

### Region of Interest (ROI) Analysis

Because we were mainly interested in activity in the thalamus region and in the subcortical areas known to be involved in tremor, in the “Tremor” protocol we restricted the analysis to regions of interest (ROIs) by applying two anatomy-based masks to the analysis. The first mask included only Thalamus, Putamen, Caudate, Cerebellum and Brainstem, excluding cortical areas (Circuit mask); the second mask was a thalamus-only mask. Anatomical regions were identified on each patient’s own T1 MRI scan using Freesurfer imaging analysis suite (http://surfer.nmr.mgh.harvard.edu, Figure S3).

## Results

At the time of evaluation all patients had moderate to severe postural tremor in the left arm (non-operated side) and minimal or no postural tremor on the right arm (operated side). We also scored tremor in the same arm position used during scanning – arm stretched, hand in pronation, wrist and fingers extended – with ETRS items 5–6 (Table 1). Left arm tremor was detected in all patients in the typical frequency range for ET (3 to 8 Hz); there was no detectable tremor in the right arm muscles used for the analysis.

### “Movement” Protocol: Analysis of Voluntary Movement

The group analysis for the “Movement” protocol (voluntary movements) performed with the block-only design, showed activity in the contralateral motor cortex and supplementary motor area, and ipsilateral cerebellum for both conditions LM and RM (Figure S4). The same analysis performed with the EMG regressor showed similar results. This confirms that the EMG regressor was able to detect brain activity related to voluntary muscle contractions.

### “Tremor” Protocol: Analysis of Tremor

#### Circuit mask

**Figure 2:**
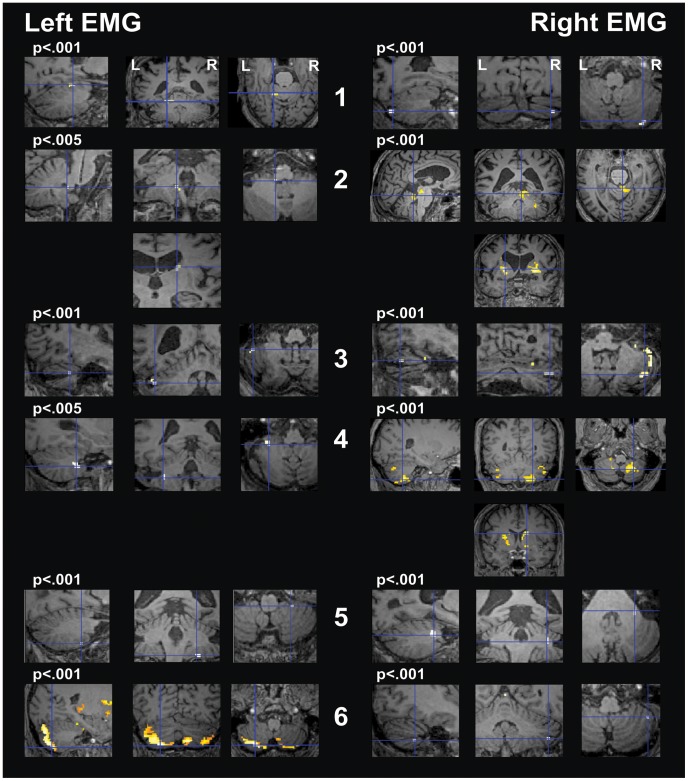
Single-subject analysis for protocol “Tremor” with the “Circuit mask”. The right hemisphere is represented on the right (“neurological view”). SPM t-contrasts, superimposed on the subjects’ own T1, are shown at a threshold of p<.001 uncorrected. For patient No. 2 and 4, Left EMG, no activity was seen at this threshold: for explorative purpose, these scans are shown at a threshold of p<.005 (p value indicated in the figure above the corresponding images). The crosshair points to the global maxima. Panels on the left show activity related to left EMG during left arm stretching (non-operated side). Ipsilateral cerebellar activation is present in all the patients except for patient 5. In four patients (No. 1, 3, 4, and 6) this represented the maximal activity, while in patient No. 2 the maximal activity was in the right caudate. In patient No. 5 the maximal activity was located in the right cerebellum and there was no clear activation of the left cerebellum. Panels on the right show activity related to right EMG during right arm stretching (operated side). Ipsilateral cerebellar activation is present in all patients. In patient No. 4 activity was maximal in the right caudate and in patient No. 2 in the left putamen.

Analysis in the ROIs of tremor circuitry showed an association between tremor of the left arm (non-operated side) and activity in the ipsilateral cerebellum in five of the six patients (Figure 2, Table S2). The EMG of the right arm (operated side) correlated with activity in the right cerebellum in all patients. In the group analysis, tremor of the left arm was associated with bilateral cerebellar activation. The EMG of the right arm correlated with bilateral activity in the caudate nucleus (Figure S5).

#### Thalamus mask

**Figure 3:**
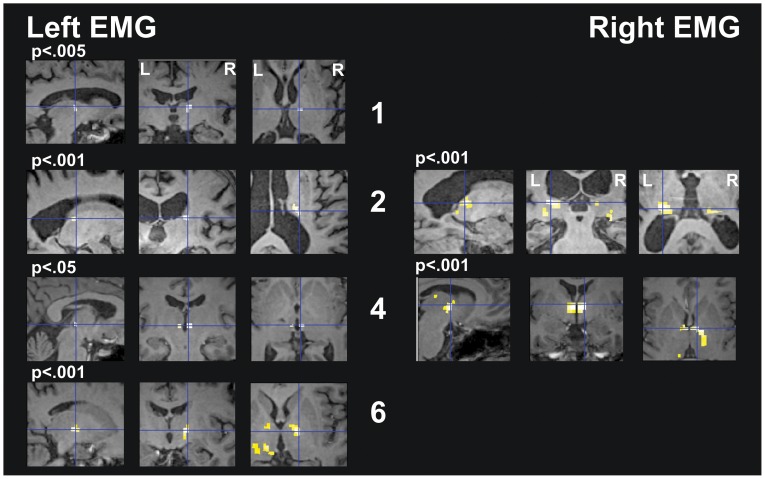
Single-subjects analysis for protocol “Tremor” with masking on the thalamus area. The right hemisphere is represented on the right (“neurological view”). SPM t-contrasts, superimposed on the subjects’ own T1, are shown at a threshold of p<.001 uncorrected. For patient No. 1 and No. 4, Left EMG, no activity was seen at this threshold: for explorative purpose these scans are shown at a threshold of p<.005 and p<.05 respectively (p value indicated in the figure above the corresponding images). The crosshair points to the global maxima. Panels on the left show activity related to left EMG during left arm stretching. Contralateral thalamic activation was present in the thalamic dorsal complex (No.1 and 6) or in the posterior thalamic region (No. 2 and 4). In patients No.3 and No. 5 there was no activation. Panels on the right show activity related to right EMG during right arm stretching. There was no thalamic activation in patients No. 1, 3, 5, and 6. In patient No. 2 and 4, bilateral thalamic activation was present, with global maxima in the left posterior thalamus (No.2) and in the right thalamic dorsal complex (No.4).

Analysis in the thalamus ROI, showed an association between left arm tremor (non-operated side) and activation in the right thalamus in most patients (No. 1, 2, 4, and 6) at variable locations, not corresponding to the Vim (Figure 3, Table S3). The EMG of the right arm (operated-side), showed no thalamic activation in most patients (No. 1, 3, 5, and 6), while in two patients bilateral thalamic activation was present. Group analysis showed no activation correlated with the left arm EMG and bilateral thalamic activation, stronger on the left side, for right arm EMG (Figure S5).

## Discussion

The first aim of our study was to provide a proof of principle of the ability of EMG-fMRI to detect BOLD activations in brain areas correlated to tremor in patients with ET.

Artifact-corrected EMG recordings clearly showed tremor bursts in the non-operated arm.

With the “Movement” protocol we could demonstrate that absolute power in the selected EMG-frequency band (1–250 Hz) indeed correlated with activation in the contralateral motor cortex and supplementary motor area, and ipsilateral cerebellum. This was in agreement with the results obtained with the conventional block-only design, and confirmed that our EMG set up was appropriate. The potential coexistence of kinetic tremor might affect results of the “Movement” protocol, especially on the left side. However, no clear increase in the tremor frequency power was identified in the frequency power spectra of the selected muscles during this protocol (Figure S2B), which suggests that the tremor component did not influence the EMG regressor.

In the “Tremor” protocol, we investigated whether EMG-fMRI could show an association of left arm tremor (non-operated side) with subcortical areas known to be involved with tremor (contralateral basal ganglia, thalamus, and cerebellum). Tremor was associated with activation in the ipsilateral cerebellum in all patients, excluding No.5. The association with cerebellar activity was also confirmed in the group analysis. This is not surprising, since studies on animal models of tremor and functional imaging studies with different modalities in ET patients suggest a major role of cerebellum in the pathophysiology of tremor [2], [21], and the cerebellum of ET patients presents pathological abnormalities [31], [32].

EMG of the right arm (operated side) was also associated with activations in the ipsilateral cerebellum in all patients. However, in the group analysis the correlation with cerebellar activation was not confirmed and rather bilateral activation of caudate nuclei was shown. This could be due to the fact that the exact spot of cerebellar activation did not overlap in all the patients. During right arm stretching the muscles selected for recording showed no tremor at clinical evaluation or on spectrograms analysis (Table 1, Figure 1), concluding that cerebellar activations were not related to the presence of residual tremor. The cerebellum is involved in the maintenance of arm posture in control subjects, as shown by conventional fMRI [1], [17], [28]. However, in an EMG-fMRI protocol similar to that used in the present study, the residual EMG power regressor did not show cerebellar activation in controls [17], [28]. This suggests that cerebellar activation in our patients was abnormal, possibly implying the presence of underlying pathological changes. The EMG in our ET patients hardly showed any fluctuations during extension of the right arm or during rest (no tremor). This could indicate that the residual EMG regressor correlates with brain activations that are present continuously. The pathological activity in the cerebellum would be independent and continuous, and thalamotomy would suppress the clinical manifestation of tremor by blocking the excitatory input to the cortex, while leaving the generating activity unaltered [33].This hypothesis is supported by PET studies in ET patients, showing that cerebellar structures and thalamus have enhanced activity, also in the absence of clinically evident tremor [4], [34].

The second aim of the present study was to test whether EMG-fMRI could predict the best thalamic spot to be used for stereotactic surgery. We hypothesized that tremor of the non-operated side would correlate with a thalamic area specular to the contralateral thalamotomy. Although in most patients left arm tremor was indeed associated with activation in the right thalamus, activation spots varied across patients and included the posterior thalamic region and dorsal nuclei, but not the Vim. In two patients there was no thalamic activation associated with tremor. The variability in thalamic activation explains also the absence of significant correlations in the group analysis and could be due to several reasons. First, patients might have a different underlying disease. It is commonly accepted that different conditions are clinically labeled as “Essential Tremor”, due to the lack of an unequivocal diagnostic test [24]. Although our patients’ cohort was extremely homogeneous with respect to disease characteristics and all patients could be classified as “Hereditary ET” [24], we cannot exclude that different pathophysiological mechanisms were involved in tremor generation in different patients. Moreover, inter-subject differences such as age, disease duration, or time from thalamotomy might play a role. Patient No. 5 presented some peculiarities in the analysis: left arm tremor was associated with activation of the contralateral cerebellum, instead that with the ipsilateral, and was not associated with the contralateral thalamus. It is of notice that this was the only patient to report permanent side effects from the surgery. We cannot exclude that a disruption of normal contralateral compensatory mechanisms might have occurred, as a result of an extension of the surgical lesion to adjacent areas.

In the patients in whom contralateral thalamic activation was indeed associated with tremor, the exact spot inside the thalamus did not correspond to the Vim. Since our protocol was designed to analyze only variations in tremor amplitude during posture, this could suggest that Vim is not involved in amplitude modulation of tremor but rather exerts a continuous and constant control on the presence of tremor, by influencing other areas of the tremor circuitry. It should also be considered that potential Vim activations could be too small to be detected, considering the present limited spatial resolution of the fMRI.

### Limitations of the Study

Our study has limitations. Although we tried to perform optimal artifact correction with a tested algorithm, and we corrected for residual artifacts and head movements with scan-nulling, we cannot totally exclude that artifacts might still, at least in part, affect the results. Similarly, different study protocols might be more appropriate. The use of the non-operated side as control, while offering the undoubtful advantage of minimizing inter-subject differences, still raises the problem of a potential influence of thalamotomy on the contralateral circuits, which could affect fMRI results.

Also, we cannot exclude that difference in performance and tremor severity across patients might account at least in part for the observed variability in results. Still, using EMG regressor to model brain activations is a better method than the conventional block design method to correct for (inter-subject) variability in motor performance and intensity of tremor.

With respect to the group analysis, the number of subjects included might be too small to show significant results on a group basis: a larger group analysis could have more power to show activity in small areas [18]. Moreover, the small sample size restricts the use of a random-effects analysis, which would allow making more robust general inferences.

Another limitation for the group analysis is that some patients showed a considerable degree of atrophy, which could affect the normalization procedure. However, this did not affect the main aim of the present study, which was to identify tremor-related areas on a single-subject basis, in order to be used for surgery planning, similarly to what is presently done also for other surgical indications.

Four of the patients included in this study were older than 75 years. Advanced age might affect the BOLD signal and fMRI results and is associated with higher degrees of atrophy. Investigating younger subjects in the future would reduce age confounding effects. However, this range of age is not unusual for the specific population included in this study, consisting of patients with advanced form of ET, who usually require functional neurosurgery later in life [8], [35], and thus it is also important to try to validate this method in this specific elderly population.

### Conclusions

EMG-fMRI is potentially useful in detecting brain activations associated with tremor in ET patients. Based on these results, however, the current EMG-fMRI protocol does not seem suitable for stereotactic target selection as yet. The methods require improvement and should be further investigated in future studies, with different cohorts of patients.

## Supporting Information

Figure S1 **EMG recordings.** Rectified EMG power recorded from the right Extensor Carpi Radialis of left and right arm during execution of “Tremor” protocol (A) and “Movement” protocol (B) for all subjects. An increase in power is clearly visible during the conditions “right arm stretching” (R stretch), “left arm stretching” (L stretch), and “both arms stretching” (B stretch) in the “tremor protocol” and during the condition “right arm movement” (R movement) and “left arm movement” (L movement) in the “Movement protocol”, as an indicator of the subjects’ performance.
(TIF)

Figure S2 **Spectrograms of EMG recorded during “Movement” protocol.** Spectrograms of the continuous simultaneous EMG recording from the Extensor Carpi Radialis of the left (top panels) and right arm (bottom panels), during the conditions rest, left movement, and right movement in patient No.1. The color bar on the right of the figure indicates power intensity going from low (deep blue) to high (red). For this protocol, analysis was done on extracted frequencies between 1 and 250 Hz (A). In B the spectrogram is zoomed between frequencies 2 and 15 Hz to show that no increase in power in the tremor frequency range was detected during execution of the movement.
(TIF)

Figure S3 **Anatomical regions of interest masks.** Anatomical masks were identified on each patient’s own T1 MRI scan using Freesurfer imaging analysis suite (example from patient No. 5). A) Thalamus mask. B) “Circuit mask” including Thalamus, Putamen, Caudate, Cerebellum and Brainstem.
(TIF)

Figure S4 **Group analysis for the protocol “Movement”.** Top panels: results obtained with a design containing only the EMG-corrected block design. Bottom panel: results obtained with a design containing only the EMG regressor. Panels on the left show activity related to condition “left arm movement”; panels on the right show activity related to condition “right arm movement”. The right hemisphere is represented on the right (“neurological view”). SPM t-contrasts for fixed-effects group analysis (6 subjects), superimposed on MNI T1 template, are shown at a threshold of p<.001 uncorrected.
(TIF)

Figure S5 **Group analysis for the protocol “Tremor”.** The right hemisphere is represented on the right (“neurological view”). SPM t-contrasts for fixed-effects group analysis (6 subjects), superimposed on MNI T1 template, are shown at a threshold of p<.001 uncorrected. Top panels show results for analysis with the “Circuit mask”. Bottom panels show results for analysis with the “Thalamus-only” mask. Panels on the left show activity related to left EMG during left arm stretching; panels on the right show activity related to right EMG during right arm stretching.
(TIF)

Table S1 **Stereotactic coordinates of the thalamotomy in mm, with respect to the Anterior Commissure.**
(DOC)

Table S2 **Clusters activated in relation to EMG power in the tremor frequency, with “circuit mask”.**
(DOC)

Table S3 **Clusters activated in relation to EMG power in the tremor frequency, with “thalamus mask”.**
(DOC)

Text S1 **Methods.**
(DOC)
