# Supplementary figures and images for: Is There a Role for Combined EMG-fMRI in Exploring the Pathophysiology of Essential Tremor and Improving Functional Neurosurgery?
Source: PLoS One. 2012 Oct 1;7(10):e46234. doi: 10.1371/journal.pone.0046234 (PMC3462183; doi:10.1371/journal.pone.0046234)

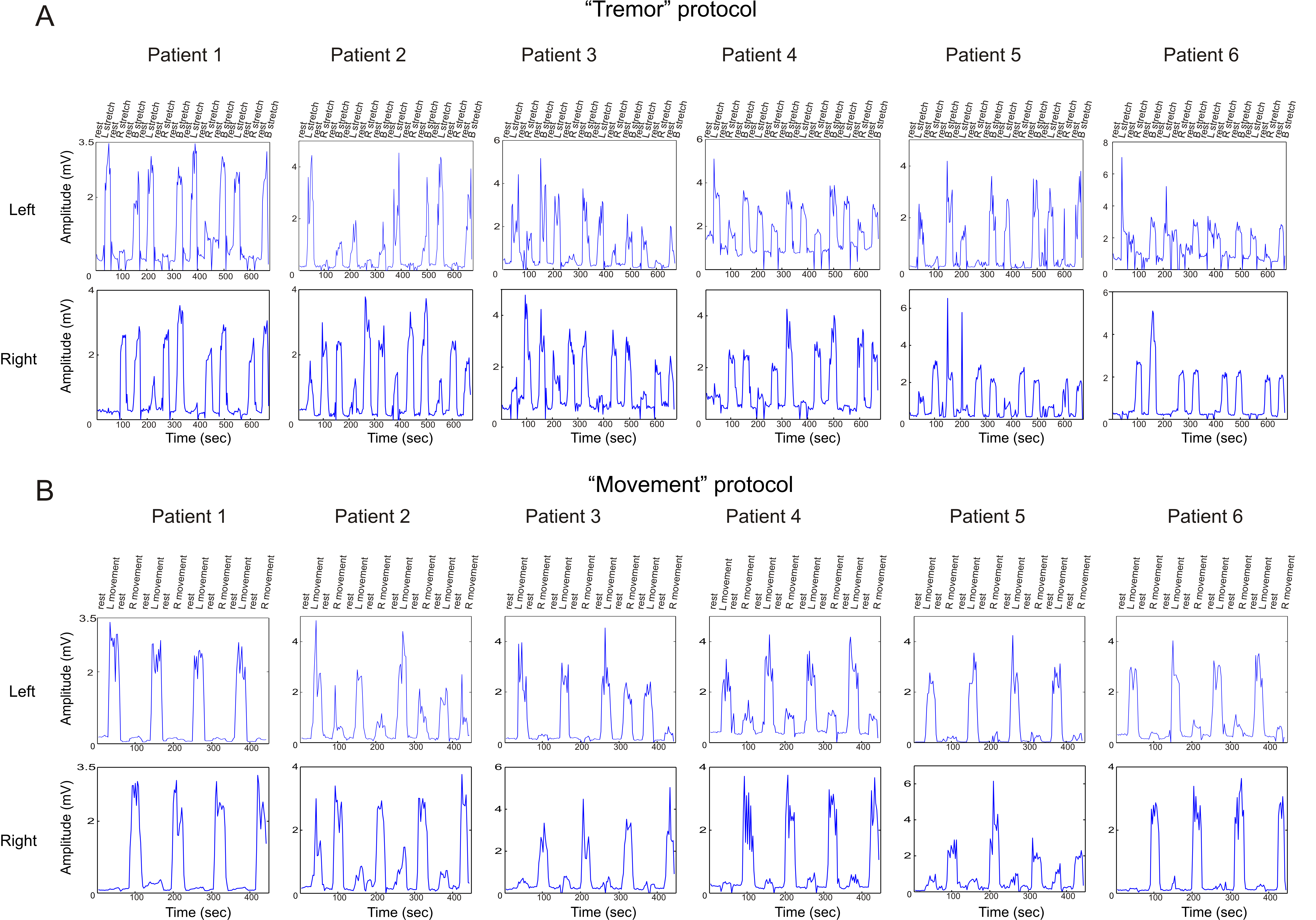

Supplement: Figure S1 — EMG recordings. Rectified EMG power recorded from the right Extensor Carpi Radialis of left and right arm during execution of “Tremor” protocol (A) and “Movement” protocol (B) for all subjects. An increase in power is clearly visible during the conditions “right arm stretching” (R stretch), “left arm stretching” (L stretch), and “both arms stretching” (B stretch) in the “tremor protocol” and during the condition “right arm movement” (R movement) and “left arm movement” (L movement) in the “Movement protocol”, as an indicator of the subjects’ performance. (TIF) [file pone.0046234.s001.tif]

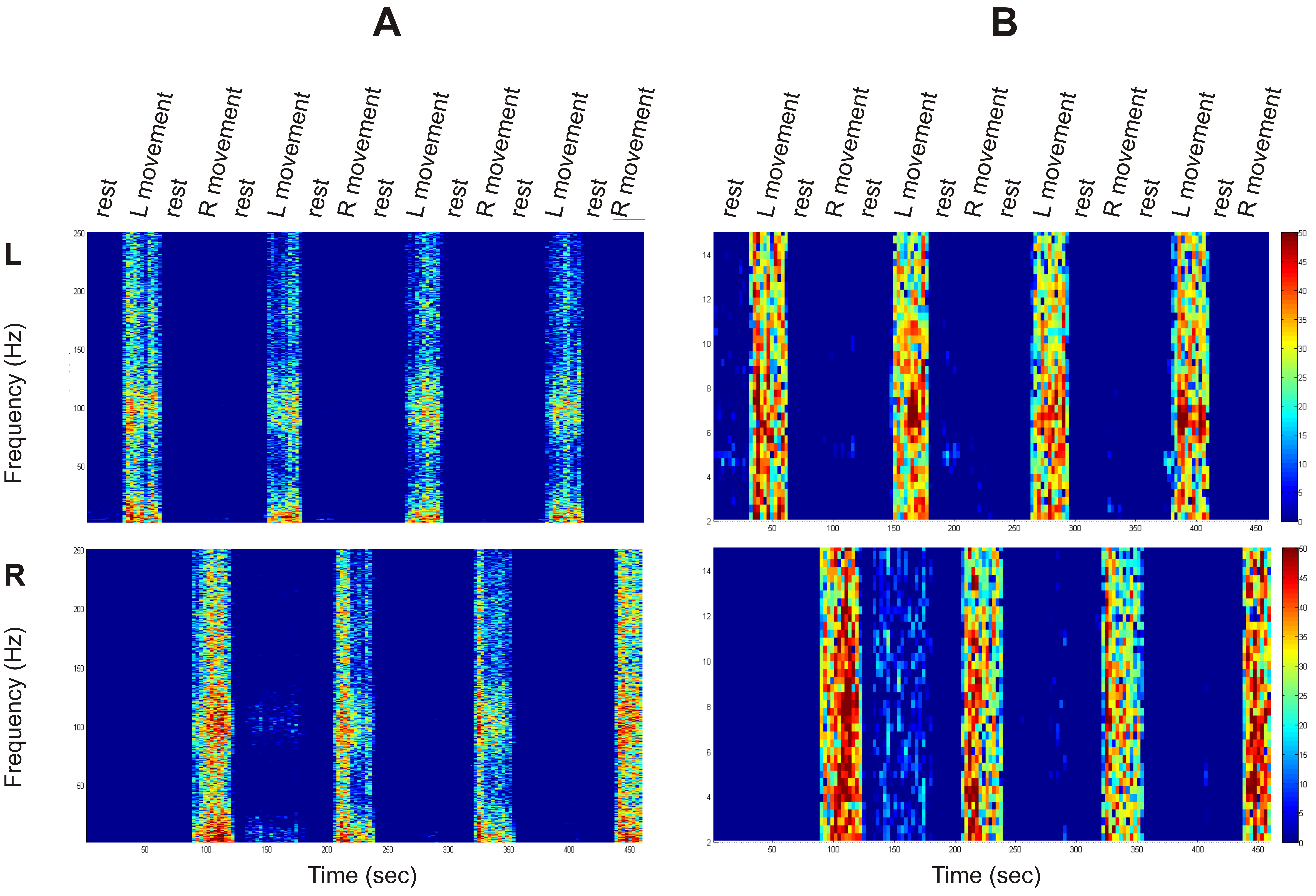

Supplement: Figure S2 — Spectrograms of EMG recorded during “Movement” protocol. Spectrograms of the continuous simultaneous EMG recording from the Extensor Carpi Radialis of the left (top panels) and right arm (bottom panels), during the conditions rest, left movement, and right movement in patient No.1. The color bar on the right of the figure indicates power intensity going from low (deep blue) to high (red). For this protocol, analysis was done on extracted frequencies between 1 and 250 Hz (A). In B the spectrogram is zoomed between frequencies 2 and 15 Hz to show that no increase in power in the tremor frequency range was detected during execution of the movement. (TIF) [file pone.0046234.s002.tif]

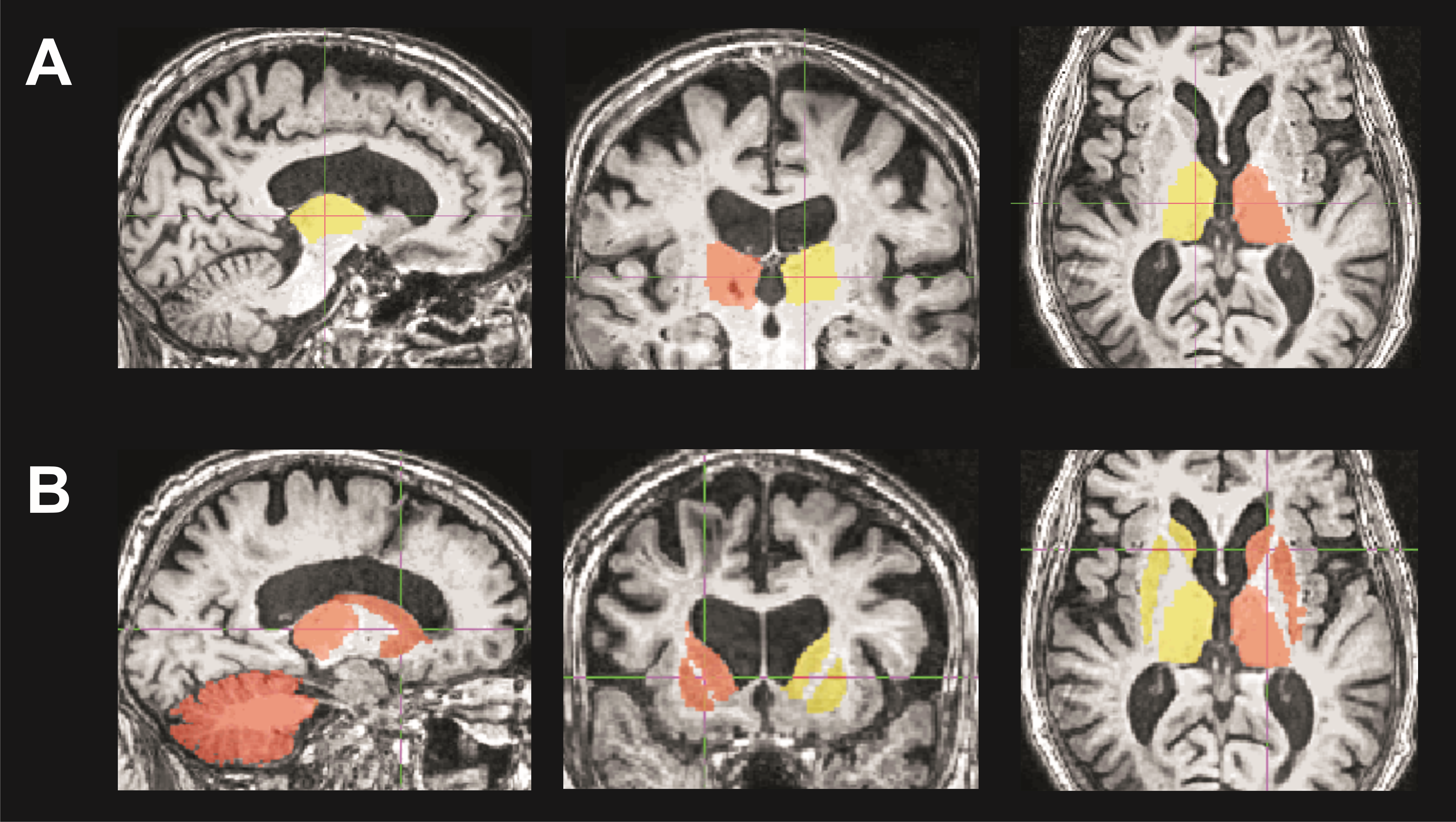

Supplement: Figure S3 — Anatomical regions of interest masks. Anatomical masks were identified on each patient’s own T1 MRI scan using Freesurfer imaging analysis suite (example from patient No. 5). A) Thalamus mask. B) “Circuit mask” including Thalamus, Putamen, Caudate, Cerebellum and Brainstem. (TIF) [file pone.0046234.s003.tif]

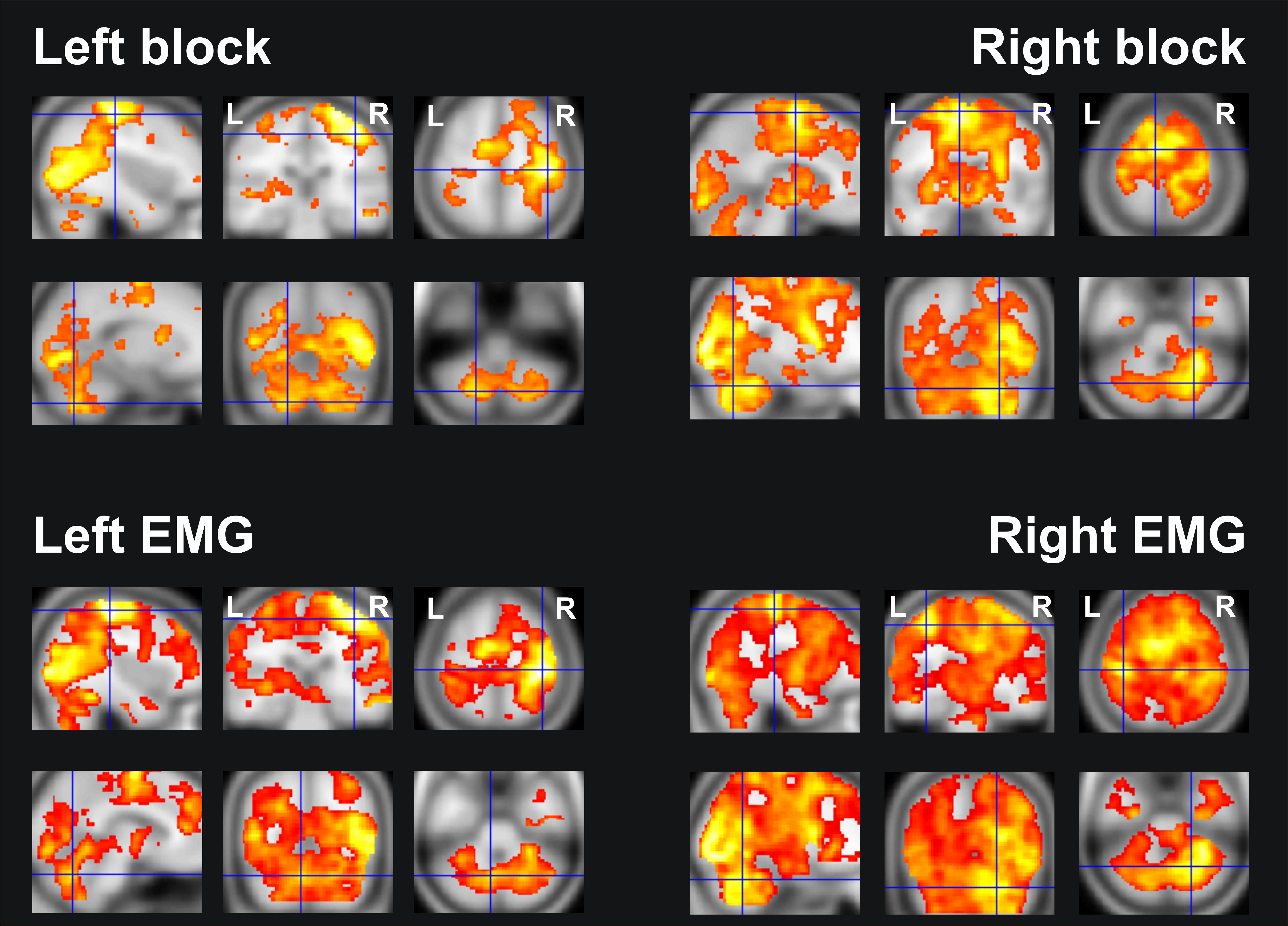

Supplement: Figure S4 — Group analysis for the protocol “Movement”. Top panels: results obtained with a design containing only the EMG-corrected block design. Bottom panel: results obtained with a design containing only the EMG regressor. Panels on the left show activity related to condition “left arm movement”; panels on the right show activity related to condition “right arm movement”. The right hemisphere is represented on the right (“neurological view”). SPM t-contrasts for fixed-effects group analysis (6 subjects), superimposed on MNI T1 template, are shown at a threshold of p<.001 uncorrected. (TIF) [file pone.0046234.s004.tif]

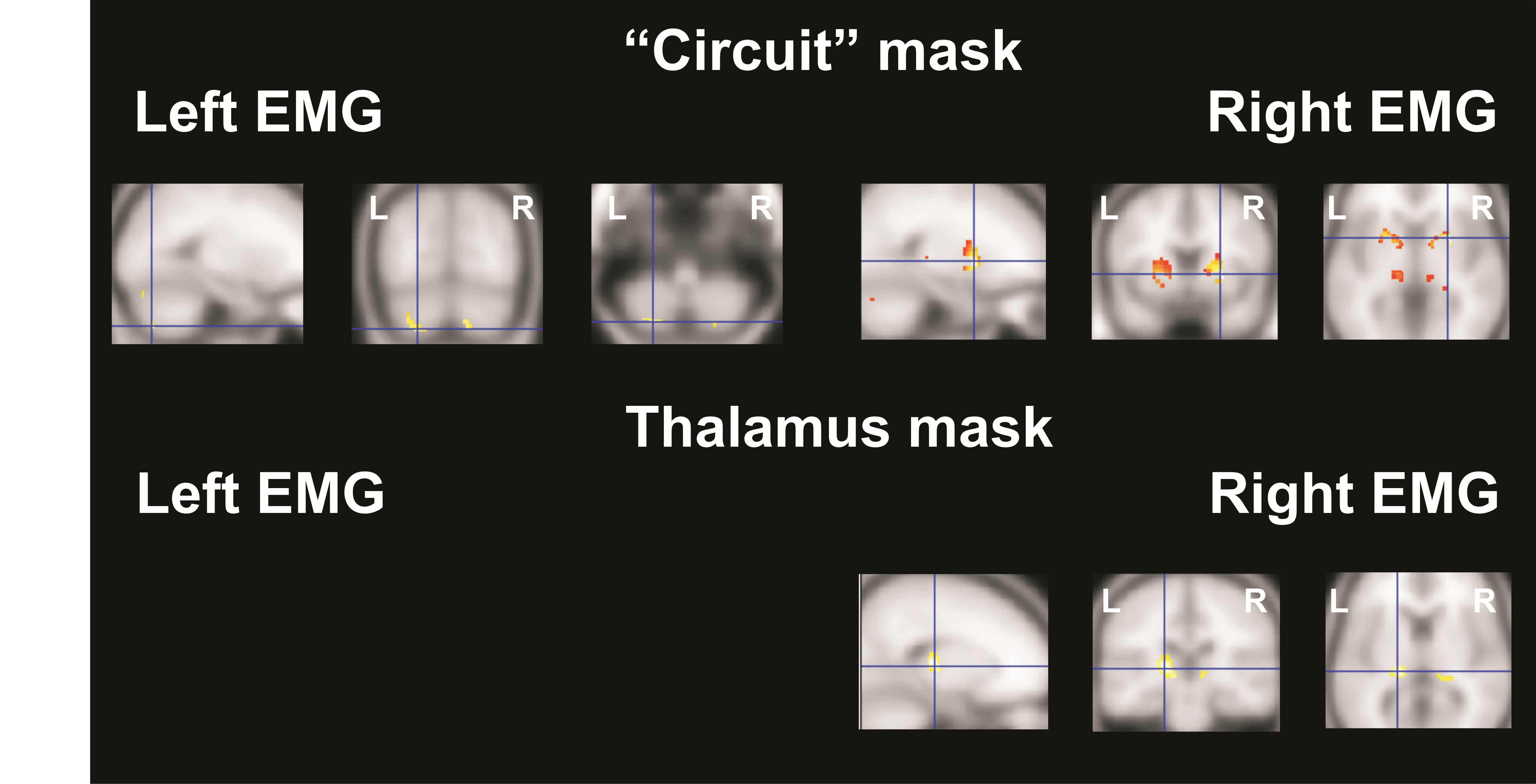

Supplement: Figure S5 — Group analysis for the protocol “Tremor”. The right hemisphere is represented on the right (“neurological view”). SPM t-contrasts for fixed-effects group analysis (6 subjects), superimposed on MNI T1 template, are shown at a threshold of p<.001 uncorrected. Top panels show results for analysis with the “Circuit mask”. Bottom panels show results for analysis with the “Thalamus-only” mask. Panels on the left show activity related to left EMG during left arm stretching; panels on the right show activity related to right EMG during right arm stretching. (TIF) [file pone.0046234.s005.tif]
