# Supplementary material for: Is There a Role for Combined EMG-fMRI in Exploring the Pathophysiology of Essential Tremor and Improving Functional Neurosurgery?
Source: PLoS One. 2012 Oct 1;7(10):e46234. doi: 10.1371/journal.pone.0046234 (PMC3462183; doi:10.1371/journal.pone.0046234)
Supplement: Table S1 — Stereotactic coordinates of the thalamotomy in mm, with respect to the Anterior Commissure. (DOC) [file pone.0046234.s006.doc]

**Supplementary Table S1** Stereotactic coordinates of the thalamotomy in mm, with respect to the Anterior Commissure.

| **Patient** | **X^a^** | **Y^b^** | **Z^c^** | **Length AC-PC line (mm)** |
| --- | --- | --- | --- | --- |
| **1** | 13 | 17 | 0 | 25 |
| **2** | 16 | 14 | 0 | 24 |
| **3** | 17 | 16 | 0 | 25 |
| **4** | 11 | 14 | +2 | n.a. (FM-PC = 22.5) |
| **5** | 15 | 18 | 0 | n.a. (FM-PC = 27) |
| **6** | 15 | 16 | 0 | 24 |
| **Average** | 14.5 ± 2.2 | 15.8 ± 1.6 | 0.3 ± 0.8 | 24.5 ± 0.6 |

**^a^**For X coordinates, positive numbers indicate locations to the left of the Anterior Commissure. **^b^**For Y coordinates, positive numbers indicate posterior to the Anterior Commissure. **^c^**For Z coordinates, positive sign indicates below the Anterior Commissure. AC, anterior commissure; FM, foramen of Monro; n.a., not available; PC, posterior commissure.
