# Supplementary material for: Is There a Role for Combined EMG-fMRI in Exploring the Pathophysiology of Essential Tremor and Improving Functional Neurosurgery?
Source: PLoS One. 2012 Oct 1;7(10):e46234. doi: 10.1371/journal.pone.0046234 (PMC3462183; doi:10.1371/journal.pone.0046234)
Supplement: Table S2 — Clusters activated in relation to EMG power in the tremor frequency, with “circuit mask”. (DOC) [file pone.0046234.s007.doc]

**Supplementary Table S2:** Clusters activated in relation to EMG power in the tremor frequency, with “circuit mask”

|  | Activations related to left arm EMG (non-operated side) | | | Activations related to right arm EMG (operated side) | | |
| --- | --- | --- | --- | --- | --- | --- |
| **Patient** | **Region of activation** | **Z score** | **No. of voxels in cluster** | **Region of activation** | **Z score** | **No. of voxels in cluster** |
| **1** | L Cerebellum | 4.59 | 12 | R Cerebellum | 3.65 | 4 |
| **2** | R Caudate  L Cerebellum | 3.44  2.86 | 16  2 | L Putamen  R Cerebellum | 5.59  4.31 | 92  69 |
| **3** | L Cerebellum | 3.90 | 4 | R Cerebellum | 4.12 | 9 |
| **4** | L Cerebellum | 2.88 | 5 | R Caudate  R Cerebellum | 5.99  4.92 | 192  90 |
| **5** | R Cerebellum | 3.45 | 2 | R Cerebellum | 3.36 | 4 |
| **6** | L Cerebellum | 7.44 | 533 | R Cerebellum | 3.56 | 1 |

EMG, electromyography; L, left; R, right.
