# Supplementary material for: Is There a Role for Combined EMG-fMRI in Exploring the Pathophysiology of Essential Tremor and Improving Functional Neurosurgery?
Source: PLoS One. 2012 Oct 1;7(10):e46234. doi: 10.1371/journal.pone.0046234 (PMC3462183; doi:10.1371/journal.pone.0046234)
Supplement: Table S3 — Clusters activated in relation to EMG power in the tremor frequency, with “thalamus mask”. (DOC) [file pone.0046234.s008.doc]

**Supplementary Table S3** Clusters activated in relation to EMG power in the tremor frequency, with “thalamus mask”

|  | Activations related to left arm EMG (non-operated side) | | | Activations related to right arm EMG (operated side) | | |
| --- | --- | --- | --- | --- | --- | --- |
| **Patient** | **Region of activation** | **Z score** | **No. of voxels in cluster** | **Region of activation** | **Z score** | **No. of voxels in cluster** |
| **1** | R dorsal thalamic complex | 2.84 | 3 | - | - | - |
| **2** | R posterior thalamus | 3.59 | 7 | L posterior thalamus | 4.28 | 45 |
| **3** | - | - | - | - | - | - |
| **4** | R posterior thalamus | 2.08 | 2 | R dorsal thalamic complex | 4.74 | 104 |
| **5** | **-** | - | - | - | - | - |
| **6** | R dorsal thalamic complex | 5.16 | 20 | - | - | - |

EMG, electromyography; L, Left; R, right.
