# Supplementary material for: Is There a Role for Combined EMG-fMRI in Exploring the Pathophysiology of Essential Tremor and Improving Functional Neurosurgery?
Source: PLoS One. 2012 Oct 1;7(10):e46234. doi: 10.1371/journal.pone.0046234 (PMC3462183; doi:10.1371/journal.pone.0046234)
Supplement: Text S1 — Methods. (DOC) [file pone.0046234.s009.doc]

## Supplementary text S1

## METHODS

## Surgical procedure

Unilateral thalamotomies were performed contralateral to the most affected side. Stereotactic surgery was performed as described previously using ventriculography.[1] In one more recently operated patient, stereotactic frame-based three-dimensional MRI was used for targeting. Final Vim coordinates for each patient, based on preoperative targeting and adjusted after intraoperative test-stimulation, are given in Supplementary Table S1. Intraoperative low and high-frequency test stimulation was carried out in 2-mm steps starting 6-8 mm above the target with a macroelectrode (2.1 × 4.0 mm bare tip). Radio-frequency thermo-lesions (70-80° Celsius, 60 seconds) were made with the same electrode used for test stimulation at one or two adjacent electrode positions with the best therapeutic effect.

## fMRI acquisition

Functional images were acquired on a 3-Tesla MRI scanner (Intera, Phillips Healthcare, Best, The Netherlands) equipped with a SENSE 6-channel head receive coil. Foam padding was used to minimize head motion. For fMRI a T2* weighted echo planar imaging (EPI) sequence was acquired with the following parameters: repetition time (TR) = 2.9 s, echo time (TE) = 30 ms, Flip angle = 80°, SENSE factor = 2.4, 96 x 95 matrix, field of view (FOV) = 220 x 220 x 150 mm, slice thickness = 3.0 mm, no inter-slice gap, voxel size = 2.3 x 2.3 x 3 mm, with 50 interleaved axial slices, in interleaved mode covering the whole brain. Two independent protocols were run. In total 160 EPI volumes during 464 s of scanning for the “Movement” protocol and 240 EPI volumes during 696 s of scanning for the “Tremor” protocol were acquired.

In addition, an isotropic T1-weighted 3D anatomical image of the whole brain was obtained (TR = 9 ms, TE = 3.5 ms, SENSE-factor = 2.5, 256 x 231 matrix, FOV = 256 x 232 x 170 mm, 170 slices, voxel size = 1 mm³, sagittal slice orientation).

## EMG acquisition

EMG data were recorded with an MRI-compatible EEG amplifier (SD MRI 64, MicroMed, Treviso, Italy) and an MRI-compatible EMG cable unit providing 16 Ag/AgCl surface electrodes configured for eight bipolar EMG derivations. A reference and a ground electrode were positioned respectively above the left and right ulnar styloid process. Electrode wires were twisted to reduce gradient artifacts and contained current-limiting electrodes of 12 kOhm. The skin was scrubbed with an abrasive gel and the EMG electrodes were attached using a plaster and conductive electrolyte gel. EMG was recorded at both arms from the extensor carpi radialis (ECR) and flexor carpi radialis (FCR) in all patients and, in addition, from the two other arm muscles which contributed most to tremor in each patient. EPI-onset information was transmitted by the MRI-scanner with a 5V TTL signal and inserted as volume-markers into the EMG data. The EMG sampling rate was 1024 Hz.

## EMG processing

EMG data processing was performed offline using Matlab (version 7.8.0 (R2009a), The Mathworks, Natick, MA). EMG data were converted into EEGLAB data format. To remove artifacts due to interaction with the magnetic field of the scanner, as well as irregular volume and slice-artifacts induced by limb motion, an adapted fMRI artifact reduction algorithm was used [2]. The quality of the EMG signal after artifact correction was visually inspected.

After artifact correction, the signal was smoothed by Hilbert-transformation, to reduce the number of outliers. Subsequently it was split into TR-based epochs, according to volume onset times and durations to allow for EMG-fMRI correlations per scan volume. Then a high-pass digital 5^th^ order Butterworth filter of 25 Hz was applied to each epoch to remove slow movement artifacts. The signal was subsequently rectified by taking the absolute value, in order to recover low frequency information [3]. Each epoch was then transformed into frequency domain using a Fast Fourier transformation. For the “Movement” protocol, we applied frequency extraction from 1 to 250 Hz (Supplementary Figure 1. In the figure, for illustration purpose only the spectrogram between frequencies 2 and 15 Hz is shown). For the “Tremor” protocol, the tremor frequency was identified and extracted, based on individual frequency power spectra (Table 1, Figure 1). Then, for each volume, the sum of the absolute EMG power in the selected frequency range was calculated. This resulted in a vector with one value per volume.

For the “Movement” protocol vectors were created from the absolute EMG power of the whole recordings of each muscle of the left and right arm. For the “Tremor” protocol, three vectors were created from the EMG of each muscle: “EMG-LS”, consisting of the absolute EMG power during stretching of the left arm and zeros elsewhere, “EMG-RS”, consisting of absolute EMG power during stretching of the right arm and zeros elsewhere, and “EMG-BS”, consisting of absolute EMG power during stretching of both arms, and zeros elsewhere.

The EMG vectors were then convolved with the haemodynamical response function (HRF) and scaled by dividing the absolute power by the SD, to make it comparable across subjects and sessions. In order to make the EMG-derived vectors and the block-derived vectors independent in the “Tremor” protocol, we applied Gram-Schmidt orthogonalization to the EMG vectors [4].

## fMRI analysis

fMRI data were analyzed with the Statistical Parametric Mapping software (SPM8, Wellcome Department of Cognitive Neurology, London, UK; [www.fil.ion.ucl.ac.uk/spm](http://www.fil.ion.ucl.ac.uk/spm)). Slice-timing correction and realignment was applied to the functional images. Images were spatially smoothed with an isotropic 8-mm full-width at half maximum (FWHM) Gaussian kernel and co-registered to T1. Data were analyzed for each patient separately at the first level, on a voxel-by-voxel basis in the context of the general linear model. The artifact-corrected EMG was used to detect the real timing of the task performance. This information was used to correct the block design with real onset and offset information (EMG-corrected block design). Head motion (translation and rotation) was determined for each EPI sequence with the slice timing correction step in SPM. The three translational parameters were used to derive a velocity parameter for each volume by calculating the movement per scan. In order to correct for excessive head movement, we excluded from the first level design all the functional scans that corresponded to a value of acceleration greater than 0.4 mm/scan by means of “scan-nulling” regressors [5]. EPI scans corresponding to residual MR artifacts on the EMG signal, especially at the beginning and end of active conditions, were also excluded by scan-nulling. For group analysis, functional images were normalized to the standard ‘Montreal Neurological Institute’ space (MNI standard space, [www.bic.mni.mcgill.ca/cgi/icbm_view](http://www.bic.mni.mcgill.ca/cgi/icbm_view)). A fixed-effects approach was used, with a threshold of p <.001 [6]. The functional t-maps were then overlaid on the T1 MNI template.

Reference List

1. Schuurman PR, Bosch DA, Bossuyt PM, Bonsel GJ, van Someren EJ, de Bie RM, Merkus MP, Speelman JD (2000) A comparison of continuous thalamic stimulation and thalamotomy for suppression of severe tremor. N Engl J Med 342: 461-468. 10.1056/NEJM200002173420703 [doi].

2. van der Meer JN, Tijssen MA, Bour LJ, van Rootselaar AF, Nederveen AJ (2010) Robust EMG-fMRI artifact reduction for motion (FARM). Clin Neurophysiol 121: 766-776. S1388-2457(10)00006-4 [pii];10.1016/j.clinph.2009.12.035 [doi].

3. Myers LJ, Lowery M, O'Malley M, Vaughan CL, Heneghan C, St Clair GA, Harley YX, Sreenivasan R (2003) Rectification and non-linear pre-processing of EMG signals for cortico-muscular analysis. J Neurosci Methods 124: 157-165. S0165027003000049 [pii].

4. van Rootselaar AF, Renken R, de Jong BM, Hoogduin JM, Tijssen MA, Maurits NM (2007) fMRI analysis for motor paradigms using EMG-based designs: a validation study. Hum Brain Mapp 28: 1117-1127. 10.1002/hbm.20336 [doi].

5. Lemieux L, Salek-Haddadi A, Lund TE, Laufs H, Carmichael D (2007) Modelling large motion events in fMRI studies of patients with epilepsy. Magn Reson Imaging 25: 894-901. S0730-725X(07)00221-4 [pii];10.1016/j.mri.2007.03.009 [doi].

6. Woods RP (1996) Modeling for intergroup comparisons of imaging data. Neuroimage 4: S84-S94. S1053-8119(96)90058-0 [pii];10.1006/nimg.1996.0058 [doi].
